# When the Belly Looks Okay but Is Actually Not: A Case of Hepatoblastoma in a Well-Appearing Child

**DOI:** 10.7759/cureus.61480

**Published:** 2024-06-01

**Authors:** Margaret A Uchefuna, Imoh L Ebong, Lamyaa Mohamed, Vanessa Ordonez, Gladys Laroche

**Affiliations:** 1 Department of Pediatrics, NYC Health + Hospitals/Woodhull Medical Center, New York, USA

**Keywords:** alpha fetoprotein, chemotherapy\, surgical resection, pediatric liver tumors, hepatoblastoma

## Abstract

Hepatoblastoma is one of the pediatric tumors with genetic and intrauterine risk factors. It is typically asymptomatic at diagnosis, at which time most patients have metastasis to the lungs and are in an advanced stage of liver disease. We report an interesting case of a 13-month-old child who presented with a one-month history of abdominal distention. A review of the systems was unremarkable but a physical examination revealed a well-appearing child with abdominal distention, normal vital signs, and an abdominal mass. Abdominal imaging revealed a well-defined heterogeneously-enhancing mass arising from the right hepatic lobe and laboratory results were consistent with a diagnosis of hepatoblastoma. The mass was resected and the patient underwent chemotherapy with continued follow-up management. We shed light on pediatric hepatoblastoma and its clinical presentation, pathology, and laboratory and imaging findings, to aid clinicians in diagnosing the condition correctly.

## Introduction

The annual incidence of hepatoblastoma in the United States is approximately 100 new cases, and the condition is often diagnosed before the age of three years [1]. It is more commonly seen in extremely premature infants with very low birth weight, and the increasing survival rate of these kids over the last two decades is directly proportional to the increased incidence of hepatoblastoma. Although no heritable cause is known, the condition can be associated with familial adenomatous polyposis, which is linked to an 800-fold higher risk of developing hepatoblastoma, and other somatic overgrowth syndromes such as Aicardi syndrome, Beckwith-Wiedemann syndrome, trisomy 18, and other trisomies [2].

Embryologically, liver tumors may arise from hepatic epithelial or mesenchymal cells with disparities in morphology, which may impact their prognosis. While the whole epithelial type (containing fetal or embryonic malignant cells) predicts a more benign process, the mixed type [containing both epithelial and mesenchymal elements and associated with normal alpha-fetoprotein (AFP) levels] predicts a worse outcome. Alterations in the antigen-presenting B cell-catenin pathway and increased insulin-like growth factor 2 expression account for its pathophysiology [3]. The tumor has a predilection for the right hepatic lobe compared to the left, and the regional lymph nodes and lungs are the usual sites of metastasis [4].

The most common presentation involves a large and asymptomatic abdominal mass; however, with disease progression, non-specific symptoms like fever, weight loss, asthenia, vomiting, abdominal pain, and respiratory distress may follow. In rare cases, the presentation may involve an acute abdomen or bleeding, following trauma or spontaneous rupture [5]. An elevated AFP has a diagnostic and monitoring value. An abdominal sonogram can be performed in highly suspicious cases to characterize hepatic masses, which can help differentiate them from benign vascular lesions [6]. An abdominal CT scan or MRI can define the extent of the involvement and the potential for surgical resection while a chest CT scan helps to evaluate for lung metastasis. A biopsy of the liver tumor establishes the diagnosis [7]. 

The treatment of the condition usually involves chemotherapy and surgical resection, and 30% of cases are resectable at diagnosis. Survival rates can vary between resectable and unresectable tumors: >90% and 60% respectively; metastasis can further decrease the survival rates.

## Case presentation

The patient was a 13-month-old boy with no significant past medical history who presented with one month of abdominal distension. Feeding was normal and he had regular bowel movements with adequate wet diapers. A review of the systems showed negative results for other symptoms. Physical examination was notable for a well-appearing, active child with abdominal distension, vital signs within normal limits, and a firm, non-tender mass measuring 12 x 12 cm in its widest diameter, originating in the right abdomen but extending across the center to the left side. 

**Figure 1:**
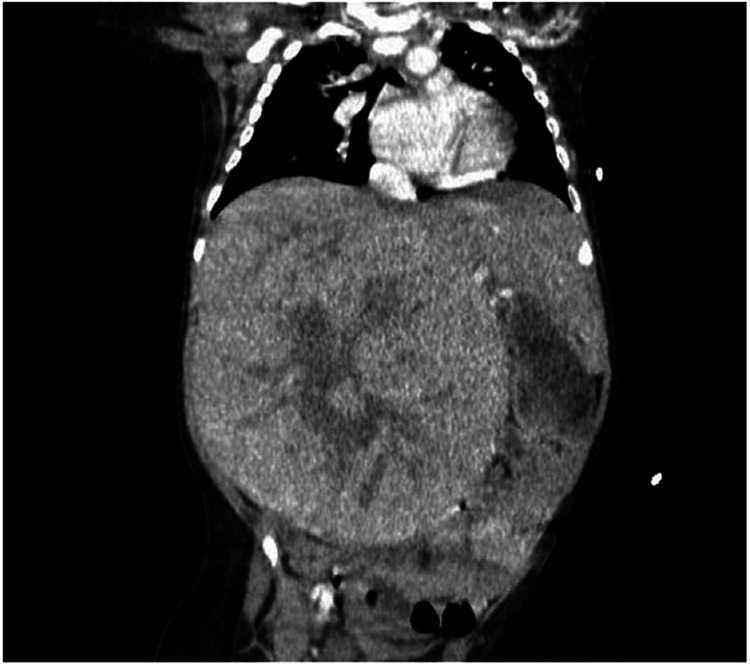
CT scan of the abdomen The image shows a well-defined heterogeneously-enhancing mass arising from the right hepatic lobe, measuring 12.4 x 11.1 x 15.9 cm, centrally hypodense with no calcifications or portal vasculature invasion CT: computed tomography

Initial complete blood count with blood film and serum electrolytes were remarkable for microcytic anemia, thrombocytosis, normal white cell count with no blasts, hyponatremia, hypophosphatemia, hypercalcemia, and normal uric acid levels. Lactate dehydrogenase and AFP levels were elevated. CT scan of the abdomen and pelvis with contrast showed a well-defined heterogeneously-enhancing mass arising from the right hepatic lobe, measuring 12.4 x 11.1 x 15.9 cm, centrally hypodense with no calcifications or portal vasculature invasion. While other organs were unremarkable, the mass had displaced the right kidney and spleen. No evidence of lung metastasis was seen on the chest CT scan. The CT scan of the abdomen showing the hepatic mass is presented in Figure 1.

**Figure 2:**
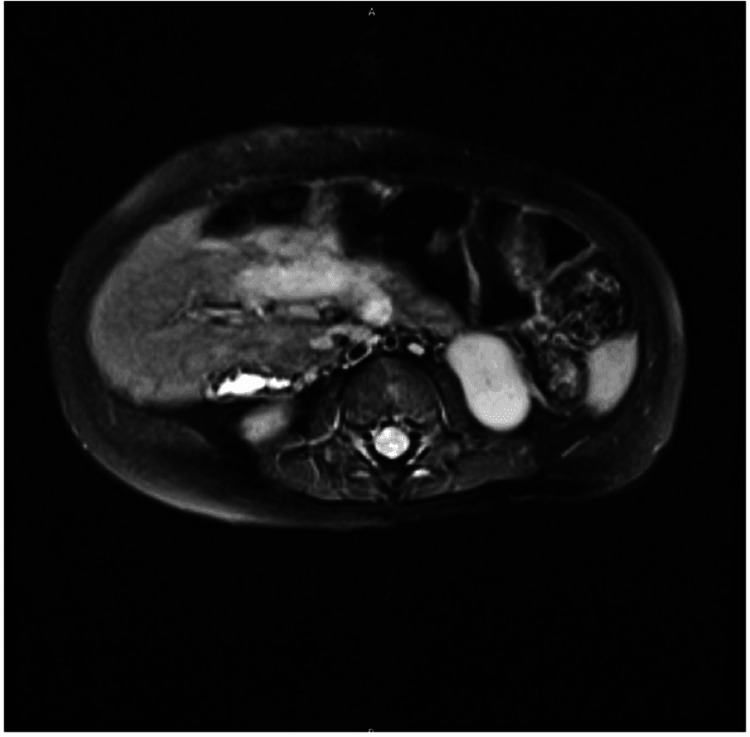
Repeat abdominal MRI with IV contrast The image shows a well-circumscribed right hepatic mass, heterogenous and somewhat nodular, measuring 6.7 x 5.6 x 4.7 cm, overall mildly T2 hyperintense and T1 hypointense. The liver capsule appears intact MRI: magnetic resonance imaging

The patient was started on nil per os alongside 5% dextrose with half normal saline + potassium chloride, allopurinol, and ferrous sulfate. Transfusion with packed red blood cells was done, and the pediatric hepatology, oncology, and surgery teams were consulted. Following a biopsy, the tumor was classified by using pretext staging as intermediate risk, class II, V+ P+. After four cycles of chemotherapy with cisplatin per the AHEP 1531 protocol, repeat abdominal MRI with IV contrast was remarkable for a well-circumscribed right hepatic mass, heterogenous and somewhat nodular, with a decrease in size to 6.7 x 5.6 x 4.7 cm, overall mildly T2 hyperintense and T1 hypointense, with an intact liver capsule (Figure 2).

Serial AFP levels showed a steady decline, and the patient subsequently underwent partial hepatectomy of the right lobe and is being followed up for completion of chemotherapy.

## Discussion

As discussed, hepatoblastomas are one of the most common childhood tumors presenting in children aged less than three years [1], as in our patient, with the most common presentation being isolated abdominal distention [5]. A unique aspect of hepatoblastoma as a pediatric tumor is its suggested correlation to prematurity, and this causes some people to believe in a link between complications of prematurity including total parenteral nutrition and oxygen therapy and the development of epithelial mutations in a developing liver [8].

Usually, some identifiable risk factors aside from prematurity include a correlation with certain genetic syndromes such as trisomy 18 and Down syndrome [8]. Our patient had been born full-term following an uneventful pregnancy, had an unremarkable nursery course, and had no identifiable syndrome to date. A biopsy was done on our patient to confirm the diagnosis of hepatoblastoma and to determine staging with the PRETEXT staging system. He was classified as stage II, V+ P+, intermediate risk, with recommendations to commence chemotherapy as per the institution’s protocol.

Some studies in the literature recommend a treatment modality with surgical resection being the mainstay of a resectable tumor, and, if the tumor is unresectable following a failed chemotherapy attempt, then liver transplantation, either partial or total should be considered [8]. Our patient received five cycles of chemotherapy with cisplatin and underwent partial hepatectomy, with notable improvement seen in abdominal MRI images and AFP levels. Currently, there are no signs of clinical metastases, and hence no indication for complete resection, which offers the optimum chance of eradication [9].

The patient’s asymptomatic presentation, coupled with his abdominal CT scan findings, was suggestive of hepatoblastoma. Clinicians should maintain a high index of suspicion when children aged less than three years present with a rapidly enlarging abdominal mass, particularly with an asymptomatic clinical picture. We also recommend further research so that less invasive modalities of treatment can be devised for pediatric hepatoblastomas.

## Conclusions

Even though hepatoblastoma is not a very common pediatric tumor, it is important to consider it in a child presenting with weight loss, abdominal mass, and decreased appetite. These clinical findings with a high index of suspicion should prompt further blood work. Hepatoblastoma in the early stages has an excellent prognosis with a five-year survival rate of 90% after resection and chemotherapy, as was done for our patient. Serum AFP is an important clinical marker for making a diagnosis and for monitoring disease progression and response to treatment. However, in some cases, AFP may not be elevated, making histology important in such cases. Nevertheless, a high index of suspicion will go a long way in helping to identify hepatoblastoma and intervene as early as possible, thereby reducing morbidity and mortality associated with the condition.
